# Harmonisation of exposure assessment: a comparison between pan-European classification FoodEx-1 and national codes

**DOI:** 10.1186/2049-3258-72-S1-P9

**Published:** 2014-06-06

**Authors:** Yasmina Akhandaf, Isabelle Sioen, Stefaan De Henauw

**Affiliations:** 1 Department of Public Health, Ghent University, Ghent 9000, Belgium

## 

A Total Diet Study (TDS) consists of selecting, collecting and analysing commonly consumed foods purchased at retail level, processing the food as for consumption, pooling the prepared food items into representative food groups, homogenizing the pooled samples and analysing them for harmful and/or beneficial chemical substances [1]. TDSs are commonly designed at national level and aim to cover the overall diet of the population, in order to assess the dietary exposure to hazardous chemical substances of interests by the population of a certain country. The selection of food items to be analysed is based on the information available in existing consumption datasets, often on national level. To assess dietary exposure, a food classification system is needed to link existing food consumption data with the analytical data obtained in the TDS. In Europe, there is a need for a harmonized TDS approach, including a harmonised exposure assessment, to make comparison between countries possible. This study assesses the practicability of FoodEx-1, a food classification system recommended by the European Food Safety Authority (EFSA), as a classification system on pan-European level and its use for exposure assessment using TDS analytical results. The comparison was made between the exposure assessment of total dioxin-like compounds using FoodEx-1 versus national codes. This was done for five European countries; Belgium, Czech Republic, the Netherlands, Spain and the UK. The main conclusion of this study was that the exposure assessment performed with FoodEx-1 did not always accurately reflect the results of the exposure assessment obtained with national codes (table 1). However, the differences observed are minimal.

**Table 1:** Percentiles of long-term exposure to dioxin-like compounds in adults living in Belgium, Netherlands, France, UK and Spain obtained via two classification systems

	Total dioxin-like compounds - Exposure (pg TEQ/kg bw/day)							
	Using national codes				Using FoodEx1 codes			

	**P50**	**P90**	**P95**	**P99**	**P50**	**P90**	**P95**	**P99**

**Belgium**	0.69	1.46	1.82	2.60	0.65	1.40	1.75	2.61

**Netherlands**	0.78	1.65	2.53	4.87	0.77	1.64	2.48	4.86

**Spain**	0.48	1.17	1.53	2.40	0.49	1.19	1.53	2.42

**UK**	0.99	1.55	1.76	2.23	0.99	1.55	1.75	2.16
